# Venous Excess Ultrasound Score Is Associated with Worsening Renal Function and Reduced Natriuretic Response in Patients with Acute Heart Failure

**DOI:** 10.3390/jcm13206272

**Published:** 2024-10-21

**Authors:** Sofya Sovetova, Kristina Charaya, Tamerlan Erdniev, Dmitry Shchekochikhin, Alexandra Bogdanova, Sergey Panov, Natalya Plaksina, Elmira Mutalieva, Natalia Ananicheva, Viktor Fomin, Denis Andreev

**Affiliations:** 1 City Clinical Hospital Named After S. S. Yudin, Kolomensky Passage 4, 115446 Moscow, Russia; 2 Department of Cardiology, Functional and Ultrasound Diagnostics, First State Moscow University Named After I.M. Sechenov, Trubetskaya 8/2, 119991 Moscow, Russia; charaya9716@gmail.com (K.C.); tamir202256@gmail.com (T.E.); agishm@list.ru (D.S.);; 3 City Clinical Hospital No. 1 Named After N.I. Pirogov, Leninsky Prospekt 8, 119049 Moscow, Russia

**Keywords:** VExUS, Doppler ultrasound, venous excess ultrasonography, acute heart failure, portal vein, hepatic vein, intra-renal vein, natriuresis, decongestion, sodium, diuretics

## Abstract

**Background:** The venous excess ultrasound score (VExUS) is used to objectify systemic venous congestion. The aim of the paper was to determine the association between VExUS grades and worsening renal function (WRF), reduced natriuretic response, diuretics resistance, and mortality in patients with acute heart failure (AHF). **Methods:** One hundred patients were included, and Doppler ultrasound of hepatic, portal, and renal veins was performed. Severity of congestion was graded using the VExUS score (grade 0, 1, 2, or 3). Sodium concentration in a spot urine sample was assessed in 2 h after the first loop diuretic administration and was adjusted for the prescribed dose of furosemide (31 mmol/40 mg). Diuretics resistance was defined as the need to double the starting dose of intravenous furosemide in 6 h. **Results:** Patients with VExUS grade 3 showed a higher incidence of WRF (OR: 11.17; 95% CI: 3.86–32.29; *p* < 0.001) and a decreased natriuretic response: a spot urine sodium content of <50 mmol/L (OR: 21.53; 95% CI: 5.32–87.06; *p* < 0.001) and an adjusted spot urine sodium content of <31 mmol/40 mg (OR: 9.05; 95% CI: 3.15–25.96; *p* < 0.001). The risk of diuretic resistance (OR: 15.31; 95% CI: 5.05–46.43; *p* < 0.001), as well as the need for inotropic and/or vasopressor support (OR: 11.82; 95% CI: 3.59–38.92; *p* < 0.001), was higher in patients with severe congestion. The hospital mortality rate increased in patients with VExUS grade 3 compared to in patients with other grades (OR: 26.4; 95% CI: 5.29–131.55; *p* < 0.001). **Conclusions:** Patients with AHF and VExUS grade 3 showed a higher risk of developing WRF, a decreased diuretic and natriuretic response, a need for inotropic and/or vasopressor support, and a poor prognosis during their hospital stay.

## 1. Introduction

Chronic heart failure is a widespread disease causing great social and economic burden due to its high prevalence, poor prognosis, and high readmission rates [1]. The majority of acute heart failure (AHF) episodes are characterized by signs and symptoms of congestion, but its severity and distribution across organs and vascular systems vary greatly among patients [2,3]. Existing clinical, instrumental, and laboratory tests have their limitations in the assessment of patients’ volume status [3,4]. In this regard, there has been an increasing interest in the search of non-invasive, fast and cost-effective methods that could identify the predominant phenotype and severity of venous congestion in order to determine the adequacy and sufficiency of diuretic therapy in a personalized manner [2,5].

In 2020, Beaubien-Souligny et al. proposed using the venous excess ultrasound score (VExUS) to assess systemic congestion in order to further predict the development of acute kidney injury (AKI) in postoperative cardiac surgery patients [6]. This grading score includes assessment of the inferior vena cava diameter (IVC) and Doppler patterns of hepatic, portal, and renal interlobular veins and is shown to be more specific for predicting AKI than any of its individual components.

The VExUS congestion grading score has still not been formally validated in patients with AHF, as there are limited data on its clinical application in this group of patients. The aim of the study was to determine whether changes in systemic venous congestion, assessed by the VExUS grading score, are associated with worsening renal function (WRF), reduced natriuretic response, diuretics resistance, the need for inotropic/vasopressor support, and poor prognosis in patients with AHF.

## 2. Materials and Methods

An observational, prospective, single-center study was conducted in a primary hospital in Moscow, Russia. The study involved patients at ≥18 years of age admitted to the cardiac intensive care unit (ICU) with AHF and requiring intravenous administration of loop diuretics. Diagnosis was based on the European Society of Cardiology (ESC) guidelines, with patients presenting with dyspnoea at rest or with minimal exertion and signs and symptoms of congestion (rales on chest auscultation, peripheral oedema, swelling of the cervical veins, hepatomegaly, ascites, and hepatojugular reflux) and N-terminal pro-B-type natriuretic peptide (NT-proBNP) of >1000 pg/mL [1].

Exclusion criteria were as follows: chronic renal replacement therapy or an estimated glomerular filtration rate (eGFR) of <15 mL/min/1.73 m^2^ (Chronic Kidney Disease Epidemiology Collaboration (CKD)-EPI) [7], acute myocardial infarction according to The Fourth Universal Definition of Myocardial Infarction [8], pulmonary embolism, sepsis (according to The Third International Consensus Definitions for Sepsis and Septic Shock (Sepsis-3)) [9], endotracheal intubation at the time of admission, pregnancy or breastfeeding, aortic dissection, liver cirrhosis Child-Pugh grade of C, active cancer, neurological or mental disease during exacerbation, and inability or refusal to sign an informed consent form.

Patients were enrolled into the study within the first 30 min upon admission to the ICU. The study design was approved by the local Ethics Committee of Sechenov University (registration No. 09-23). Patients were included after signing an informed consent form.

To assess natriuretic response, sodium concentration in a spot urine sample was measured in 2 h after the first intravenous loop diuretic administration and then was adjusted for the prescribed diuretic dose (Na: 31 mmol/40 mg of furosemide) [10]. Patients received an initial dose of diuretic after emptying the bladder, either through ureteral catheterization or spontaneous urination. The dose of intravenous furosemide was determined predominantly by the ESC position paper on diuretic therapy (starting dose: 40–80 mg in a diuretic-naïve patients or 2 times the usual home dose) [5]. The cumulative dose of loop diuretics was assessed in the period of the first 72 h of their hospital stay. Urine output volume was assessed in the first 6 and 24 h after the administration of a loop diuretics.

### 2.1. Venous Excess Ultrasound Score

**Figure 1:**
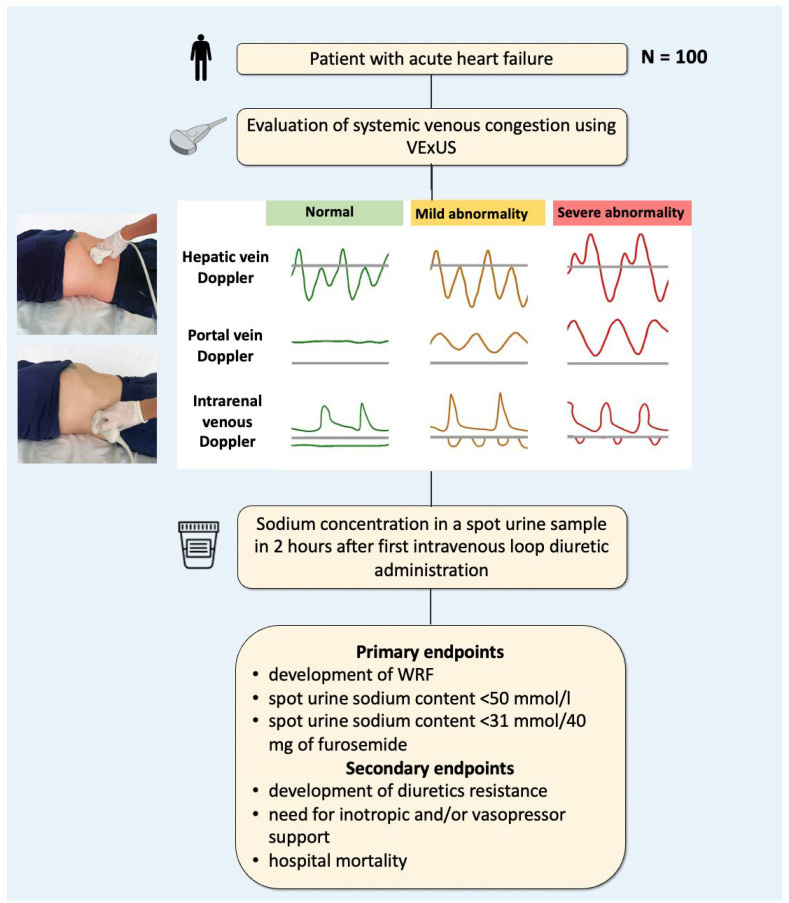
Study protocol. VExUS—venous excess ultrasound score; WRF—worsering renal function.

All the patients upon admission underwent ultrasound evaluation of organ venous congestion, which included assessment of IVC diameter and collapsibility, hepatic vein Doppler, portal vein Doppler, and intrarenal venous Doppler. Then, the VExUS score was calculated according to the previous reports [6]. Absence of congestion was determined as VExUS grade 0 (IVC < 2 cm); mild congestion represented VExUS grade 1 (IVC ≥ 2 cm with normal patterns or mild abnormalities of hepatic and renal blood flows); moderate congestion was consistent with VExUS grade 2 (IVC ≥ 2 cm with severe abnormality in at least one venous flow pattern), and VExUS grade 3 was defined as severe congestion (IVC ≥ 2 cm and severe abnormalities in multiple patterns) (Figure 1).

For the portal vein Doppler, a pulsatility index fraction of >50% and a reversal flow were considered as congestive patterns [11]. For the intrarenal venous Doppler, a discontinuous pattern with only a diastolic phase (monophasic renal blood flow) was considered as severe renal congestion [6].

### 2.2. Study Endpoints

The primary endpoints were as follows: (1) the development of WRF, defined as oligoanuria (diuresis rate < 0.5 mL/kg/hour for 6 h) [12], and an increase of serum creatinine of >26.5 μmol/L within 48 h or 50% from baseline creatinine within a week [13]; (2) spot urine sodium concentration of <50 mmol/L in 2 h after the first loop diuretic administration; (3) spot urine sodium concentration with adjustment for the prescribed initial dose of diuretic (<31 mmol/40 mg of furosemide).

The secondary endpoints included the following points: (1) the development of diuretics resistance (defined as the need to double the starting dose of intravenous furosemide (40–80 mg in a diuretic-naïve patients or 2 times the usual home dose) in 6 h without adding a different class of diuretic agents) [5,14]; (2) need for inotropic and/or vasopressor support; (3) hospital mortality for any cause.

### 2.3. Statistical Analysis

Data were analyzed using the SPSS software version 26. Continuous variables were expressed as the mean and standard deviation if normally distributed or as the median with 25th–75th percentiles if not. Continuous variables between the four grades of the VExUS score were compared using the Kruskal–Wallis test followed by the Bonferroni test for between-group comparisons. Categorical variables were expressed using frequency and percentage. The association between the VExUS grades and specified endpoints was analyzed by the Fisher’s exact test.

## 3. Results

From March 2022 to November 2023, 100 patients were enrolled. Baseline characteristics are presented in Table 1. The mean age was 73.5 (64–81) years, and 40% were women. A total of 75% had underlying chronic coronary disease, 20% were diagnosed with chronic obstructive pulmonary disease, 48% presented atrial fibrillation at admission, and 30% had diabetes mellitus. De novo AHF was recorded in 51% of patients. Regarding the echocardiographic findings on admission, the median left ventricular EF was 32 (25–49)%; in 25% patients, EF was preserved. According to the laboratory data, the median level of NT-proBNP level was 6115 (3669–12,477) pg/mL and the mean eGFR was 56.95 ± 25.5 mL/min/1.73 m^2^ (Table S1 in the Supplementary Materials).

VExUS grade 0 (no congestion) was observed in 36 patients, VExUS grade 1 (mild congestion) was observed in 26 patients, VExUS grade 2 (moderate congestion) was observed in 12 patients, and VExUS grade 3 (severe congestion) was observed in 26 patients. A congestive portal venous pattern with pulsation flow more than 50% (including flow inversion) was present in 33 patients. Regarding the intrarenal veins, normal (continuous) renal blood flow was observed in 61 patients, biphasic renal blood flow was observed in 21 patients, and monophasic renal blood flow was observed in 18 patients.

Within the first hour of admission, patients were prescribed 80 (80–100) mg of intravenous furosemide. During their hospital stay (7.5 (5–9) days), all the patients received diuretic therapy without discontinuation. The median of sodium concentration in a single urine test obtained in 2 h after the start of diuretic therapy was 114 (90–131) mmol/L. The median diuresis amount obtained within 6 h from the start of the treatment was 1300 (750–2000) mL, and that obtained within 24 h from the start of the treatment was 3000 (2250–3775) mL.

During hospitalization, WRF developed in 37 patients. A reduced natriuretic response with sodium concentration of less than 50 mmol/L in a spot urine sample was detected in 15 patients, while with an adjustment for the prescribed furosemide dose (31 mmol Na/40 mg of furosemide) that was detected in 23 patients. Resistance to diuretic therapy (the need to double the initial dose of furosemide in 6 h) was observed in 23 patients. Some patients (17) required inotropic and/or vasopressor support. Thirteen patients died during the hospital stage, 11 required mechanical ventilation, and 3 were in need for kidney replacement therapy.

### 3.1. Association Between the VExUS Grade Score and WRF

Among 37 patients with WRF, VExUS grade 0 was observed in 9 patients (24%), VExUS grade 1 was observed in 6 patients (16%), VExUS grade 2 was observed in 2 patients (5%), and VExUS grade 3 was observed in 20 patients (54%).

**Figure 2:**
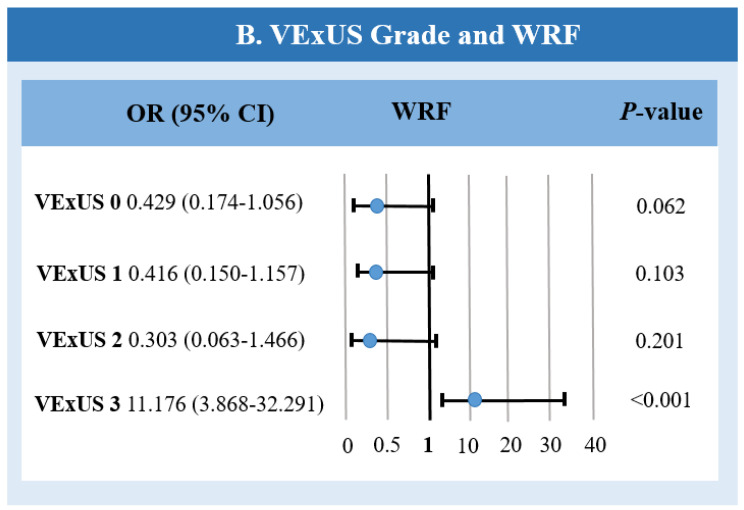
The VExUS grade and worsering renal function. CI—confidence interval; OR—odds ratio; VExUS—venous excess ultrasound score; WRF—worsering renal function.

In patients with severe congestion (VExUS grade 3), the incidence of WRF was the highest: among 26 patients in this group, WRF was developed in 20 patients (76%) (OR: 11.17; 95% CI: 3.86–32.29; *p* < 0.001), while among patients with mild to moderate congestion, there was no statistically significant increase in the risk of developing WRF (*p* < 0.001) (Figure 2).

As for renal congestion, WRF developed in all patients with monophasic renal blood flow (n = 18) (RR: 4.31; 95% CI: 2.9–6.41; *p* < 0.001), and there was a higher risk of WRF compared to continuous and biphasic patterns of renal blood flow (*p* < 0.001). It was also observed that patients with a congestive pattern of portal venous flow had a higher risk of developing WRF compared to those with a normal or slightly altered portal blood flow pattern (OR: 5.57; 95% CI: 2.25–13.78; *p* < 0.001), although with a lower incidence of WRF in this group compared to patients with a monophasic renal blood flow pattern (64% vs. 100%).

### 3.2. Association Between the VExUS and Natriuretic Response

Sodium concentrations of less than 50 mmol/L in a spot sample obtained in 2 h after the first intravenous furosemide administration was observed in 15 patients (15%). Most of these patients (n = 12) had VExUS grade 3 (OR: 21.53; 95% CI: 5.32–87.06; *p* < 0.001), and all of them demonstrated the most altered (monophasic) pattern of renal blood flow (OR: 35.29; 95% CI: 8.58–145.14; *p* < 0.001). Thirteen patients (86%) with a urine sodium concentration of <50 mmol/L had congestive portal blood flow (OR: 21.89; 95% CI: 4.53–105.69; *p* < 0.001).

A reduced natriuretic response with an adjustment for the prescribed furosemide dose (31 mmol Na/40 mg of furosemide) was also observed in patients with VExUS grade 3 (OR: 9.05; 95% CI: 3.15–25.96; *p* < 0.001), while the other grades did not demonstrate any correlation. Both congestive portal (OR: 6.39; 95% CI: 2.32–17.63; *p* < 0.001) and monophasic renal blood (OR 15.27, 95%CI: 4.5–51.81, *p* < 0.001) flow showed reduced adjusted natriuretic responses.

**Figure 3:**
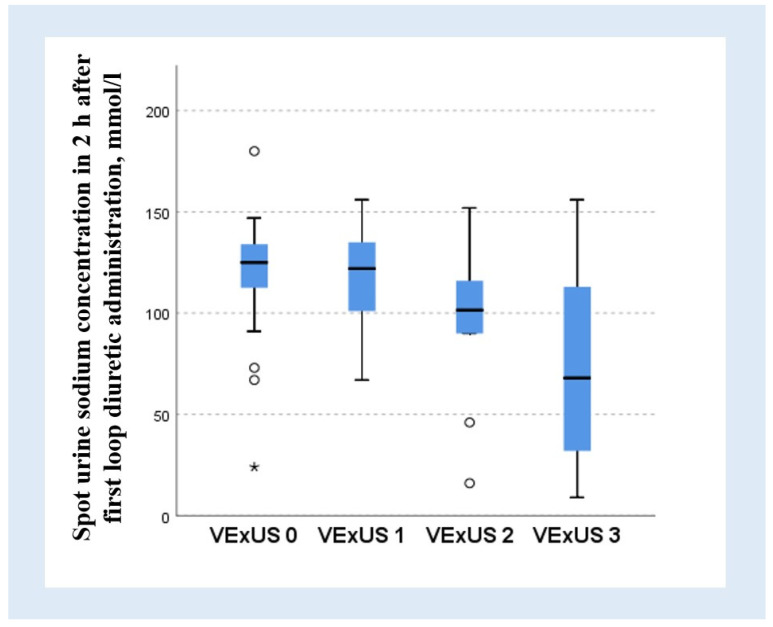
Spot urinary sodium concentration in 2 h after the first standard intravenous loop diuretic administration across different VExUS grades. The black horizontal line in the middle of the boxes is the median sodium concentration. A symbol (○) indicates outliers. A symbol (*) indicates extreme outliers. VExUS—venous excess ultrasound score.

The median sodium concentration in urine in patients with VExUS grade 0 was 125 (112.5–134) mmol/L, that with grade 1 was 122 (101–135) mmol/L, and that with grade 2 was 101.5 (90–116) mmol/L, while in patients with most severe congestion (grade 3) the sodium concentration in spot urine samples was only 68 (32–113) mmol/L and on average was decreased compared to other groups with different VExUS grades (*p* < 0.001) (Figure 3).

### 3.3. Association Between the VExUS Grade and Diuretic Resistance and the Need for Inotropic and/or Vasopressor Support

Diuretic resistance was observed in 23 patients; among them, 3 patients (13%) had VExUS grade 0, 3 patients (13%) had VExUS grade 1, VExUS grade 2 was present in only 1 patient (4%), and 16 patients (69%) had VExUS grade 3.

**Figure 4:**
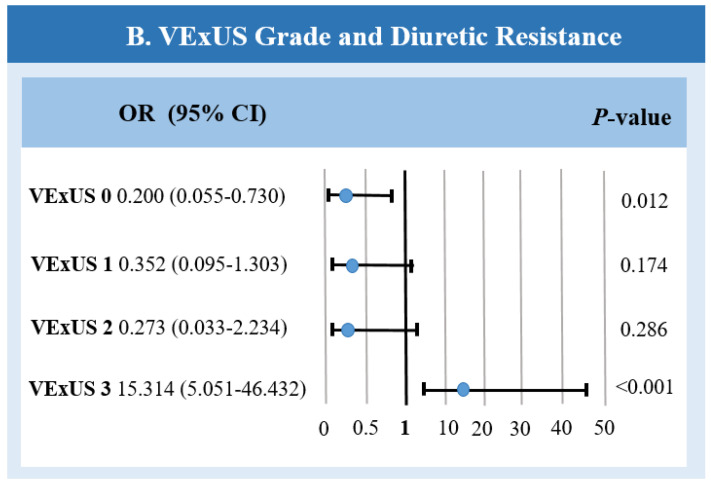
Risks of diuretic resistance in patients with different VExUS grades. CI—confidence interval; OR—odds ratio; VExUS—venous excess ultrasound score.

Patients with VExUS grade 3 were more likely to develop diuretic resistance compared to patients with other grades of venous congestion (OR: 15.31; 95% CI: 5.05–46.43; *p* < 0.001) (Figure 4).

Patients with a monophasic renal blood flow pattern showed an even higher risk of diuretic resistance (OR: 28.38; 95% CI: 7.66–105.13; *p* < 0.001), while the congestive portal blood flow pattern showed the lowest predictive value (OR: 8.06; 95% CI: 2.85–22.79; *p* < 0.001).

During their ICU stay, 17 patients (17%) required inotropic and/or vasopressor support, and patients with VExUS grade 3 were at the highest risk for this compared to the patients with mild to moderate congestion (OR: 11.82; 95% CI: 3.59–38.92; *p* < 0.001).

### 3.4. Association Between the VExUS and Hospital Mortality

A total of 13 patients (13%) died during the hospital stage; among them, VExUS grade 0 was found in 0% cases, VExUS grade 1 was found in 1 patient (7%), VExUS grade 2 was found in 1 patient (7%), and VExUS grade 3 was found in 11 patients (84%).

The risk of hospital mortality was increased in patients with VExUS grade 3 (OR: 26.4; 95% CI: 5.29–131.55; *p* < 0.001).

It was also found that patients with a monophasic pattern of renal blood flow had an even higher risk of hospital mortality (OR: 32.91; 95% CI: 7.48–144.75; *p* < 0.001) when compared to those with other patterns of intrarenal blood flow. The congestive portal blood flow compared to other portal flow patterns also showed an increased risk of this adverse event, although to a lesser extent (OR: 16.25; 95% CI: 3.33–79.07; *p* < 0.001).

## 4. Discussion

In this study, it has been shown that patients with VExUS grade 3 were at higher risk of developing WRF, diuretic resistance with a decreased natriuretic response, and hospital mortality. Doppler ultrasound of hepatic, portal, and renal veins is an easy-to-use bedside method that objectively assesses the severity of congestion in patients with AHF. Below, we will discuss some pathogenesis and clinical applications of the VExUS protocol in various clinical situations with an analysis of previous studies.

### 4.1. Worsening Renal Function

According to the results of our study, patients with VExUS grade 3, the most altered portal blood flow, and a monophasic renal blood flow were at higher risk of developing WRF, which is consistent with data from other studies. Beaubien-Souligny et al. found that severe alterations in portal and intrarenal blood flows are the markers of venous congestion and are independently associated with AKI following cardiac surgery [15]. Although changes in renal blood flow are more significantly associated with AKI, it was noted that assessment of portal blood flow is more convenient for use in clinical practice due to its simplicity. Based on other studies, a significantly lower GFR and its decrease were observed in heart failure patients with the most pathological (monophasic) intrarenal blood flow pattern [16,17,18], while the resolution of AKI correlated with improvements in portal blood flow pulsatility and in VExUS grades [19,20]. Such clinical findings may be explained by several pathophysiological mechanisms. An increased central venous pressure (CVP) appears as an afterload pressure on renal veins, causing compression of renal parenchyma and structures, which leads to a decrease in glomerular filtration. The decreased GFR has been widely studied in the context of AHF, where the increased CVP played a major role in the deterioration of renal function [21,22].

In our study, 20 of 26 patients with VExUS grade 3 developed WRF, and it is worth noting that the 6 patients, who did not demonstrate renal function deterioration, maintained a normal renal blood flow. At the same time, all the patients with a monophasic renal flow pattern acquired WRF. There was also observed that patients with a congestive portal blood flow had a higher risk of developing WRF compared to those with a normal or slightly altered portal flow pattern. Thus, according to our data and the results of some previous studies, it can be assumed that the assessment of intrarenal blood flow is the most accurate in terms of predicting deterioration in renal function. However, given the technical difficulties in intrarenal blood flow obtaining and time-consuming VExUS scoring, it seems reasonable to assess portal blood flow solely instead of predicting WRF. This idea is supported by data from other studies [15,23,24].

### 4.2. Natriuretic Response

The adverse effects of renal venous congestion on urine output volume and urine sodium excretion have been documented in previously published studies and involve a variety of pathophysiological mechanisms [25,26]. First, an increase in renal interstitial hydrostatic pressure caused by venous congestion may lead to a reduction in the driving force necessary for fluid filtration from blood plasma to Bowman’s space [26]. Second, increased lymph flow followed by elevated renal parenchymal pressure triggers the removal of interstitial proteins, thereby diminishing interstitial colloid osmotic pressure within the kidneys, which consequently promotes the reabsorption of sodium and water [26,27]. Third, renal venous congestion prompts activation of the sympathetic nervous system and the renin−angiotensin−aldosterone system, which are responsible for increased reabsorption of sodium and water in renal tubules, and also causes constriction of the renal veins [26].

Several studies and the European Society of Cardiology guidelines have demonstrated the significance of evaluating spot urine sodium concentration as an early indicator of effective diuretic therapy [5,28,29]. In our study, we found that spot urine sodium concentration obtained in 2 h after the first standard intravenous furosemide administration correlated with the severity of venous congestion (graded according to VExUS), as well as with congestive Doppler patterns in portal and renal veins. We also performed an adjustment for the prescribed diuretic dose, whereas it has been recently demonstrated that spot natriuresis per 40 mg of furosemide equivalents might perform better to assess diuretic response [10]. In our study, both (adjusted and unadjusted spot urine sodium) were associated with severe congestion (VExUS grade 3; congestive portal and monophasic renal blood flow), with a stronger association using unadjusted sodium concentrations. However, we suggested using both methods to determine diuretic response, especially to apply adjustments for furosemide if higher doses were prescribed (≥40–80 mg in a diuretic-naïve patients or ≥2 times the usual home dose). In our study, it helped us to identify a larger number of patients with a reduced natriuretic response.

As there are no common guidelines on how to assess effective diuretic and natriuretic responses, it seems reasonable to use multifactorial analysis including spot natriuresis and visual assessment of systemic congestion with the help of VExUS. Using both methods (laboratory and instrumental) may help to assess patients at high risk of WRF, diuretic resistance, mortality, and adjust treatment within the first few hours of hospitalization.

### 4.3. Diuretic Resistance

At the moment, there is a lack of studies assessing the relationship between haemodynamic alterations in venous hepatic, portal, or intrarenal blood flow patterns and diuretic resistance. A 2017 study by Nijst et al. conducted on 50 euvolemic HF patients noted that the presence of highly pulsatile renal blood flow patterns reduced the effectiveness of diuretic therapy compared to continuous renal blood flow patterns [30]. Another study involving 188 AHF patients found that those with discontinuous renal flow patterns required higher daily and cumulative furosemide doses compared to those with continuous renal venous patterns [18].

There is currently no consensus on what to define diuretic resistance (weight-derived, diuresis-derived, or spot-natriuresis-derived) [10]. We chose to define diuretic resistance based on ESC position paper on diuretic therapy as the need to double the starting dose of furosemide when one of two events was recorded: spot natriuresis of <50 mmol/L and/or a 6 h urine output of less than 100 mL/h) [5]. According to the results of our study patients with VExUS grade 3, highly pulsatile portal vein flow and monophasic renal blood flow were at higher risk of diuretics resistance. This makes us believe that VExUS could be used in addition to the bedside ultrasound point-of-care (POCUS) protocol in order to identify patients, which could be in benefit from more aggressive diuretic therapy. In addition to this, the VExUS protocol could be used to monitor efficacy of diuretic therapy, since spot sodium in the context of determining diuretic resistance is used only for the early initial assessment (the first 24 h according to ESC guidelines) [5]. This notion is reflected in several publications indicating that effective diuretic therapy improves the VExUS grade score and intrarenal blood flow patterns [18,31,32]. Although assessing VExUS over the course of hospitalization was not the purpose of our study, this may be the subject for further trials to find a new method to guide decongestive therapy.

### 4.4. Inotropes/Vasopressors

It is known that right ventricular (RV) failure is associated with increased RV, right atrial pressure, and systemic congestion. Retrograde transmission of an elevated right atrial pressure (RAP) leads to abdominal venous congestion and deterioration of renal function [33]. RV failure can also impair left ventricular filling and reduce systemic cardiac output through ventricular interdependence [34]. To restore end-organ perfusion and reduce cardiac filling pressures, noradrenaline and/or inotropes are indicated for low cardiac output and hemodynamic instability [1]. In our study, patients with VExUS grade 3 more often required inotropic or vasopressor support compared to those with mild to moderate congestion; however, we did not find data supporting these findings. At the same time, some publications have explored the interaction between right ventricular filling pressures and VExUS grade scores. Longino et al. found a strong correlation between the RAP and the VExUS grade score; VExUS grade 3 predicted an RAP of 12 mm Hg or higher with a sensitivity of 1 (95% CI: 0.69–1) and a specificity of 0.85 (95% CI: 0.71–0.94) [35]. The studies by Husain-Syed et al. and Iida et al. discovered a correlation between biphasic and monophasic renal blood flow patterns and elevated RAP (measured by cardiac catheterization) in pulmonary hypertension patients, which also has been shown to be a strong predictor of adverse clinical outcomes [17,36]. These findings show a significant association between VExUS grading and RAP across a diverse range of patients, potentially allowing for the avoidance of invasive procedures in critically ill patients and guiding decongestive therapy through ultrasound.

### 4.5. Mortality

Our study revealed a correlation between VExUS grade 3, monophasic renal blood flow, congestive portal blood flow, and hospital mortality. These findings are supported by previous research. A 2023 study on AHF patients demonstrated that VExUS grade 3 upon admission was shown as an adequate predictor of mortality. Additionally, portal and renal assessments have shown themselves as potential mortality predictors, suggesting that they could be used as the sole prognostic factor, given the ease of obtaining [12,24]. A number of studies on outpatient and hospitalized patients with chronic HF have established that the presence of intrarenal biphasic or monophasic blood flow correlates with adverse clinical events, such as death from cardiovascular diseases and readmissions for AHF [16,36,37,38].

The poor prognosis in these patients may be explained by severe venous congestion, as VExUS grade 3 and renal congestion reflect profound hypervolemia. Resolving these conditions can be difficult in the setting of reduced diuretic/natriuretic response and a higher risk of developing WRF. In this clinical setting, ultrasound examination of systemic venous flow upon admission may help stratify patients’ prognosis and identify high-risk patients eligible for additional decongestive strategies, including ultrafiltration as a bailout therapy [5].

### 4.6. Study Limitations

First, the small number of patients limited the power of the study. Second, patients in this study received therapy in the ICU department, which limits application of our results to the general population of patients with heart failure. Third, we did not have data on the dynamics of the patients’ weights during their ICU stay, since this was not the purpose of our study and also ICU patients adhere to strict bed care. Fourth, there was no strict or standardized protocol for diuretic therapy (treatment was mostly individualized according to clinical findings), which could have affected the results of the study. In the present study, it was not possible to determine the modifying effects of concomitant factors (such as diabetes mellitus, arterial hypertension, neurohormonal disorders, and ongoing inflammatory response) on the blood flow patterns through changing renal or hepatic parenchyma or function of the vessels. In this regard, further research is needed to gain a deeper understanding of the pathophysiology of the process. We did not perform an invasive hemodynamic assessment and therefore cannot establish a direct correlation between blood flow patterns and right-sided filling pressures. We did not measure intra-abdominal pressure, so its potential influence on vascular Doppler ultrasound was not assessed. Finally, the obtained venous Doppler images were not reviewed by an independent laboratory.

## 5. Conclusions

Patients with VExUS grade 3 were at higher risk of developing WRF and showed a decreased natriuretic response. This group also showed a higher incidence of diuretics resistance, a need for inotropic and/or vasopressor support as well as a poor prognosis during their hospital stay.

## Figures and Tables

**Table 1:** Patients’ baseline characteristics.

	Total (n = 100)	VExUS Grade 0 (n = 36)	VExUS Grade 1 (n = 26)	VExUS Grade 2 (n = 12)	VExUS Grade 3 (n = 26)	*p*-Value
Sociodemographic characteristic						
Age	73.5 (64–81)	73.5 (64.5–81)	73.5 (64–83)	74.5 (66.5–79)	70.5 (60–81)	*p* = 0.886
Sex (female), n (%)	40 (40)	14 (39)	11 (42)	6 (50)	9 (35)	*p* = 0.830
Body mass index, kg/m^2^	27.65 (24.85–30.1)	27.45 (25.4–32.15)	26.7 (22.8–29.9)	27.9 (26.2–29.4)	28.15 (25.9–30.1)	*p* = 0.584
Smoking, n (%)	51 (51)	14 (39)	11 (42)	6 (50)	20 (77)	*p* = 0.018
Hypertension, n (%)	96 (96)	35 (97)	25 (96)	11 (92)	25 (96)	*p* = 0.736
Diabetes mellitus, n (%)	30 (30)	12 (33)	6 (23)	5 (42)	7 (27)	*p* = 0.628
Atrial fibrillation, n (%)	62 (62)	19 (53)	16 (62)	6 (50)	21 (81)	*p* = 0.115
CAD ^a^, n (%)	75 (75)	31 (86)	21 (81)	9 (75)	14 (54)	*p* = 0.028
Previous myocardial infarction, n (%)	72 (72)	29 (81)	20 (77)	7 (58)	16 (62)	*p* = 0.243
Valvular disease, n (%)	6 (6)	1 (3)	2 (8)	1 (8)	2 (8)	*p* = 0.687
HFpEF, n (%)	25 (25)	7 (19)	4 (15)	4 (33)	10 (39)	*p* = 0.195
NICM, n (%)	8 (8)	0 (0)	4 (15)	0 (0)	4 (15)	*p* = 0.028
COPD, n (%)	20 (20)	7 (19)	5 (19)	2 (17)	6 (23)	*p* = 0.969
Clinical characteristic						
Systolic blood pressure, mm Hg	141.73 ± 34.35	155.44 ± 29.68	145.77 ± 35.68	139.5 ± 24.95	119.7 ± 33.17	*p* < 0.001
Heart rate, bpm	95.5 (79.5–111.5)	100 (83–117)	94 (80–110)	86 (72.5–102.5)	100 (79–115)	*p* = 0.212
Rhythm of AF at admission, n (%)	48 (48)	15 (42)	11 (42)	4 (33)	18 (69)	*p* = 0.9
Signs and symptoms of congestion ^b^	1.33 (1–2.66)	1 (1–1.33)	1.33 (1–2)	2.66 (2.17–2.66)	2.66 (2–2.66)	*p* < 0.001
SOFA	2 (1–4)	1 (1–2.5)	1 (1–3)	2 (1–4)	4 (3–5)	*p* < 0.001
Laboratory data						
NT-proBNP, pg/mL	6115 (3669–12,477.5)	3800 (3295.5–8175.5)	4518 (3140.5–7248)	12,883 (5171.5–17,871)	9338 (6612–15,429)	*p* = 0.01
Serum potassium, mmol/L	3.87 ± 0.76	3.67 ± 0.57	4.14 ± 0.82	3.81 ± 0.9	3.9 ± 0.81	*p* = 0.106
Serum sodium, mmol/L	137 (134–139)	138 (136.2–140)	136 (134–139)	135.5 (133.5–140.5)	134.1 (130–139)	*p* = 0.096
Hyponatremia <135 mmol/L, n (%)	33 (33)	7 (19)	8 (31)	6 (50)	12 (46)	*p* = 0.087
Lactate, mmol/L	2.3 (1.79–3.4)	2.45 (1.85–3.4)	2.1 (1.4–2.5)	2.43 (2–3.1)	2.4 (2–4.6)	*p* = 0.225
Hemoglobin, g/L	129.68 ± 22.54	133.44 ± 17.36	127.85 ± 22.83	128.42 ± 24.06	126.88± 27.95	*p* = 0.663
Anemia, n (%)	38 (38)	8 (22)	12 (46)	4 (33)	14 (54)	*p* = 0.061
Leukocytes × 10^9^/L	9.25 (7.2–11.55)	9.7 (8.2–11.7)	8.65 (7.2–11.5)	8.75 (7–10.9)	9.45 (7.7–12)	*p* = 0.836
CRP, mg/ml	12.46 (2.6–37.05)	14.18 (2.65–39.85)	11.15 (0.8–25.38)	4.87 (0–26.8)	13.8 (6.27–54)	*p* = 0.286
Albumin, g/L	36.67 ± 5.61	38.43 ± 4.71	37.08 ± 4.71	35.32 ± 7.1	34.84 ± 6.29	*p* = 0.087
Creatinine at admission, µmol/L	106.9 (80.95–149.5)	94.9 (80.8–116.2)	86.95 (70–107.8)	118.05 (76.75–138)	166.3 (120–248)	*p* < 0.001
Maximum creatinine level, µmol/L	125 (98–170.5)	115.5 (101.5–140.5)	102.5 (87.9–138)	119.1 (91.7–141.5)	213 (133–299)	*p* < 0.001
eGFR at admission, mL/min/1.73 m^2^	56.95 ± 25.5	62.23 ± 22.64	67.23 ± 26.2	57.33 ± 21.79	39.19 ± 22.02	*p* < 0.001
eGFR < 60 mL/min/1.73 m^2^ at admission	59 (59)	19 (53)	11 (42)	8 (67)	21 (81)	*p* = 0.028
Urea, mmol/L	8.2 (6.75–11.5)	7.6 (6.15–9.5)	7.55 (5.2–11.1)	9.4 (7.2–13.9)	16.05 (8.2–24.1)	*p* < 0.001
Total bilirubin, mg/dL	15.85 (9.75–22.3)	11.25 (7.35–17.4)	15.2 (11.7–25.7)	16 (12.95–24.75)	20.3 (15.2–29.6)	*p* = 0.005
ALT, IU/L	25.35 (17.25–46.5)	21.95 (16.85–33.75)	24.75 (12.4–35.4)	40.4 (19.4–59.8)	26.85 (18.4–81)	*p* = 0.182
AST, IU/L	30.35 (21.6–53.15)	24.8 (18.45–33.5)	29.5 (20–53)	30 (22.5–59.25)	45 (30.5–87)	*p* < 0.001
LDL, mmol/L	2.425 (1.9–3.19)	2.62 (1.91–3.4)	2.35 (2–2.85)	2.18 (1.8–2.49)	2.61 (1.69–3.09)	*p* = 0.668
Sodium in spot urine sample, mmol/L	114 (90–131)	125 (112.5–134)	122 (101–135)	101.5 (90–116)	68 (32–113)	*p* < 0.001
Sodium in spot urine <50 mmol/L	15 (15)	1 (3)	0 (0)	2 (17)	12 (48)	*p* < 0.001

AF—atrial fibrillation; ALT—alanine transaminase; AST—aspartate transaminase; CAD—coronary artery disease; COPD—chronic obstructive pulmonary disease; CRP—C-reactive protein; eGFR—estimated glomerular filtration rate; HFpEF—heart failure with preserved ejection fraction; LDL—low-density lipoprotein; NT-proBNP—N-terminal pro-B-type natriuretic peptide; SOFA—sequential organ failure assessment. ^a^ CAD was defined as obstructive coronary artery lesions according to previous coronary angiogram, previous coronary artery stenting, and previous coronary artery bypass surgery. ^b^ Signs/symptoms: dyspnea (1 point), oedema (absence of oedema—0 points; ankles—0.33 points; up to knees—0.66 points; above the knees—1 point), and jugular venous distension (1 point). Values were presented as the median [25th–75th percentiles], the mean ± standard deviation, or as n (%).

## Data Availability

The authors confirm that the data supporting the findings of this study are available from the corresponding author upon reasonable request.

## Supplementary Materials

The following supporting information can be downloaded at: https://www.mdpi.com/article/10.3390/jcm13206272/s1, Table S1: Patient baseline characteristics.
