# Supplementary material for: Venous Excess Ultrasound Score Is Associated with Worsening Renal Function and Reduced Natriuretic Response in Patients with Acute Heart Failure
Source: J Clin Med. 2024 Oct 21;13(20):6272. doi: 10.3390/jcm13206272 (PMC11508279; doi:10.3390/jcm13206272)
Supplement: Supplementary file 1 [file jcm-13-06272-s001.zip › jcm-3243121-supplementary.pdf]

Table S1. Patient baseline characteristics

	Total (n=100)	VExUS grade 0 (n=36)	VExUS grade 1 (n=26)	VExUS grade 2 (n=12)	VExUS grade 3 (n=26)	P-value	Congestive portal flow (n=33)	Monophasic renal flow (n=18)
Sociodemographic characteristic								
Age	73.5 [64 -81]	73.5 [64.5-81]	73.5 [64-83]	74.5 [66.5-79]	70.5 [60-81]	p=0.886	69 [62-81]	68 [60-81]
Sex (female), n (%)	40 (40)	14 (39)	11 (42)	6 (50)	9 (35)	p=0.830	11 (33)	6 (33)
Body mass index, kg/m^2	27.65 [24.85-30.1]	27.45 [25.4-32.15]	26.7 [22.8-29.9]	27.9 [26.2-29.4]	28.15 [25.9-30.1]	p=0.584	27.7 [25.9-29.3]	27.85 [25.9-29.7]
Smoking, n (%)	51 (51)	14 (39)	11 (42)	6 (50)	20 (77)	**p=0.018**	24 (73)	15 (83)
Hypertension, n (%)	96 (96)	35 (97)	25 (96)	11 (92)	25 (96)	p=0.736	31 (94)	18 (100)
Diabetes mellitus, n (%)	30 (30)	12 (33)	6 (23)	5 (42)	7 (27)	p=0.628	11 (33)	6 (33)
Atrial fibrillation, n (%)	62 (62)	19 (53)	16 (62)	6 (50)	21 (81)	p=0.115	24 (73)	16 (89)

CAD[a], n (%)	75 (75)	31 (86)	21 (81)	9 (75)	14 (54)	**p=0.028**	19 (58)	10 (56)
Previous myocardial infarction, n (%)	72 (72)	29 (81)	20 (77)	7 (58)	16 (62)	p=0.243	19 (58)	12 (67)
Valvular disease, n (%)	6 (6)	1 (3)	2 (8)	1 (8)	2 (8)	p=0.687	3 (9)	2 (11)
HFpEF, n (%)	25 (25)	7 (19)	4 (15)	4 (33)	10 (39)	p=0.195	13 (39)	7 (39)
NICM, n (%)	8 (8)	0 (0)	4 (15)	0 (0)	4 (15)	**p=0.028**	4 (12)	4 (22)
COPD, n (%)	20 (20)	7 (19)	5 (19)	2 (17)	6 (23)	p=0.969	8 (24)	4 (22)
Clinical characteristic								
Systolic blood pressure, mm Hg	141.73 ± 34.35	155.44± 29.68	145.77 ± 35.68	139.5 ± 24.95	119.73 ± 33.17	**p<0.001**	123.73 ± 30.99	114.83 ± 33.63
Heart rate, bpm	95.5 [79.5-111.5]	100 [83-117]	94 [80-110]	86 [72.5-102.5]	100 [79-115]	p=0.212	95 [74-110]	100 [66-110]
Rhythm of AF at admission, n (%)	48 (48)	15 (42)	11 (42)	4 (33)	18 (69)	p=0.9	19 (58)	14 (78)
Signs and	1.33 [1-	1 [1-	1.33 [1-	2.66	2.66 [2	**p<0.001**	2.66 [2 -	2.66 [2 -

symptoms of congestion[b]	2.66]	1.33]	2]	[2.17-2.66]	-2.66]		2.66]	2.66]
SOFA	2 [1-4]	1 [1-2.5]	1 [1-3]	2 [1-4]	4 [3-5]	**p<0.001**	4 [2-5]	5 [4-6]
Laboratory data								
NT-proBNP, pg/mL	6115 [3669-12477.5]	3800 [3295.5-8175.5]	4518 [3140.5-7248]	12883 [5171.5-17871]	9338 [6612-15429]	**p=0.01**	9737 [5471-16800]	10052 [8378-16034]
Serum potassium, mmol/L	3.87 ± 0.76	3.67 ± 0.57	4.14 ± 0.82	3.81 ± 0.9	3.9 ± 0.81	p=0.106	3.87 ± 0.8	4.08 ± 0.68
Serum sodium, mmol/L	137 [134-139]	138 [136.2-140]	136 [134-139]	135.5 [133.5-140.5]	134.1 [130-139]	p=0.096	135 [130-139]	131 [126-136]
Hyponatremia <135 mmol/L, n (%)	33 (33)	7 (19)	8 (31)	6 (50)	12 (46)	p=0.087	15 (46)	11 (61)
Lactate, mmol/L	2.3 [1.79-3.4]	2.45 [1.85-3.4]	2.1 [1.4-2.5]	2.43 [2-3.1]	2.4 [2-4.6]	p=0.225	2.43 [1.65-4.1]	2.7 [2-4.7]
Hemoglobin, g/L	129.68 ± 22.54	133.44 ± 17.36	127.85 ± 22.83	128.42 ± 24.06	126.88 ± 27.95	p=0.663	127.82 ± 27.92	130.39 ± 23.41

Anemia, n (%)	38 (38)	8 (22)	12 (46)	4 (33)	14 (54)	p=0.061	16 (49)	9 (50)
Leukocytes x 10^9/L	9.25 [7.2-11.55]	9.7 [8.2-11.7]	8.65 [7.2-11.5]	8.75 [7-10.9]	9.45 [7.7-12]	p=0.836	9.2 [7.1-11.6]	9.45 [8.2-11.6]
CRP, mg/ml	12.46 [2.6-37.05]	14.18 [2.65-39.85]	11.15 [0.8-25.38]	4.87 [0-26.8]	13.8 [6.27-54]	p=0.286	13.4 [4.22-56]	36.4 [12.6-104]
Albumin, g/L	36.67 ± 5.61	38.43 ± 4.71	37.08 ± 4.71	35.32 ± 7.1	34.84 ± 6.29	p=0.087	35.07 ± 6	34.48 ± 5.9
Creatinine at admission, μmol/l	106.9 [80.95-149.5]	94.9 [80.8-116.2]	86.95 [70-107.8]	118.05 [76.75-138]	166.3 [120-248]	**p<0.001**	142 [115.1-205]	189.5 [123-263]
Maximum creatinine level, μmol/l	125 [98-170.5]	115.5 [101.5-140.5]	102.5 [87.9-138]	119.1 [91.7-141.5]	213 [133-299]	**p<0.001**	182 [123.65-275]	250 [189-320]
eGFR at admission, mL/min/1.73 m^2	56.95 ± 25.5	62.23 ± 22.64	67.23 ± 26.2	57.33 ± 21.79	39.19 ± 22.02	**p<0.001**	43.82 ± 23.91	33.72 ± 17.41
eGFR < 60 mL/min/1.73, n (%) m^2 at	59 (59)	19 (53)	11 (42)	8 (67)	21 (81)	**p=0.028**	26 (79)	16 (89)

admission								
Urea, mmol/L	8.2 [6.75-11.5]	7.6 [6.15-9.5]	7.55 [5.2-11.1]	9.4 [7.2-13.9]	16.05 [8.2-24.1]	**p<0.001**	13.6 [7.9-23.6]	19.4 [11.2-24.5]
Total bilirubin, mg/dL	15.85 [9.75-22.3]	11.25 [7.35-17.4]	15.2 [11.7-25.7]	16 [12.95-24.75]	20.3 [15.2-29.6]	**p=0.005**	19.9 [14.1-29.6]	21.85 [18.7-52]
ALT, IU/L	25.35 [17.25-46.5]	21.95 [16.85-33.75]	24.75 [12.4-35.4]	40.4 [19.4-59.8]	26.85 [18.4-81]	p=0.182	31.9 [18.8-81]	27.5 [18.4-92.9]
AST, IU/L	30.35 [21.6-53.15]	24.8 [18.45-33.5]	29.5 [20-53]	30 [22.5-59.25]	45 [30.5-87]	**p<0.001**	47 [29-71.5]	52.65 [34.7-94]
LDL, mmol/L	2.425 [1.9-3.19]	2.62 [1.91-3.4]	2.35 [2-2.85]	2.18 [1.8-2.49]	2.61 [1.69-3.09]	p=0.668	2.55 [1.81-3]	2.63 [1.69-3.11]
Sodium in spot urine sample, mmol/L	114 [90-131]	125 [112.5-134]	122 [101-135]	101.5 [90-116]	68 [32-113]	**p<0.001**	88 [32.5-120.5]	35 [29-85]
Sodium in spot urine <50 mmol/L, n (%)	15 (15)	1 (3)	0 (0)	2 (17)	12 (48)	**p<0.001**	13 (41)	11 (65)

Medical treatment								
ACEi/ARB, n (%)	76 (76)	31 (86)	23 (89)	12 (100)	10 (39)	**p<0.001**	17 (52)	4 (22)
β-Blocker, n (%)	54 (54)	24 (67)	14 (54)	9 (75)	7 (27)	**p=0.006**	13 (39)	3 (17)
Loop diuretics, n (%)	100 (100)	36 (100)	26 (100)	12 (100)	26 (100)		33 (100)	18 (100)
Cumulative dose of loop diuretics, mg	240 [160-420]	230 [160-300]	200 [160-320]	260 [180-470]	460 [240-620]	**p<0.001**	380 [240-570]	540 [360-640]
Duration of diuretic therapy	7.5 [5-9]	7.5 [6-9]	7 [5-8]	8 [6-9]	8.5 [5-12]	**p=0.527**	8 [5-11]	8 [3-12]
Aldosterone antagonist, n (%)	56 (56)	16 (44)	19 (73)	10 (83)	11 (42)	**p=0.013**	18 (55)	7 (39)
Inotropic and/or vasopressor support, n (%)	17 (17)	1 (3)	2 (8)	2 (17)	12 (46)	**p<0.001**	12 (36)	11 (61)
Mechanical	11 (11)	0 (0)	1 (4)	1 (8)	9 (35)	**p<0.001**	9 (27)	8 (44)

ventilation, n (%)								
Renal replacement therapy, n (%)	3 (3)	0 (0)	0 (0)	0 (00)	3 (12)	p=0.069	3 (9)	3 (17)
Echocardiography								
LVEF, %	32 [25-49]	34 [25-48]	30 [25-35]	31 [22-57]	39 [24-55]	p=0.764	34 [25-57]	40 [23-55]
LVEDD	5.6 [4.8-6.2]	5.8 [4.8-6.2]	5.6 [4.9-6.2]	5.5 [4.9-6.3]	5.3 [4.6-6.3]	p=0.847	5.4 [4.7-6.3]	5.6 [4.5-6.6]
LVESD	4.2 [3.2-5.6]	4.4 [3.3-5.3]	4.7 [3.5-5.8]	4.1 [3.3-5.3]	3.8 [3.1-5.8]	p=0.898	3.9 [3.2-5.6]	4.1 [3.1-6.1]
E/e’	18.55 ± 7.39	17.78 ± 7.36	19.75 ± 6.12	19.64 ± 5.23	17.73 ± 9.74	p=0.697	18.14 ± 8.82	13.63 ± 10.95
LVOT VTI, cm	13.05 [9.85-17.45]	14.6 [12-17.9]	13 [9.5-17.9]	13.4 [8.7-15.5]	11 [9-13.35]	p=0.186	11.45 [9-13.5]	9 [8.7-11]
TAPSE, mm	15.76 ±	17.79 ±	15.23 ±	17 ± 2.9	12.4 ±	**p<0.001**	13.3 ± 2.81	12 ± 1.5

	3.57	2.79	3.59		2.21			
TAPSE < 17 mm, n (%)	52 (52)	13 (39)	16 (62)	4 (36)	19 (95)	**p<0.001**	22 (85)	13 (100)
Moderate or severe TR, n (%)	67 (67)	15 (42)	18 (69)	12 (100)	22 (89)	**p<0.001**	28 (88)	16 (89)
ePASP, mm Hg	47.88 ± 16.62	36.06 ± 11.94	47.62 ± 14.24	61.08 ± 11.12	58.42 ± 15.37	**p<0.001**	58.33 ± 15.85	57.22 ± 13.59
RV S’, cm/s	11.34 ± 3.33	12.85 ± 3.29	11.29 ± 2.87	11.81 ± 4.27	9.05 ± 1.99	**p<0.001**	9.11 ± 2.15	8.91 ± 2.26
IVC > 2 cm, n (%)	64 (64)	0 (0)	26 (100)	12 (100)	26 (100)	**p<0.001**	33 (100)	18 (100)
Course of the disease								
WRF, n (%)	37 (37)	9 (25)	6 (23)	2 (17)	20 (77)	**p<0.001**	21 (64)	18 (100)
Diuretic resistance, n (%)	23 (23)	3 (8)	3 (12)	1 (8)	16 (62)	**p<0.001**	16 (49)	14 (78)
Urine output in 6 h, ml	1300 [750-2000]	1300 [1000-2000]	1800 [1200-2500]	1500 [1100-2200]	400 [200-1500]	**p<0.001**	800 [300-1700]	350 [200-500]

Urine output in 24 h, ml	3000 [2250-3775]	3150 [2500-3900]	3350 [2600-4200]	3400 [2800-6050]	2000 [800-3200]	**p=0.003**	2600 [1300-3300]	1500 [800-2100]
Hospital mortality, n (%)	13 (13)	0 (0)	1 (4)	1 (8)	11 (42)	**p<0.001**	11 (33)	10 (56)

ACEi = angiotensin-converting-enzyme inhibitors; AF = atrial fibrillation; ALT = alanine transaminase; ARB = angiotensin II receptor blockers; AST = aspartate transaminase; CAD = coronary artery disease; COPD = chronic obstructive pulmonary disease; CRP = C-reactive protein; E/e′ = ratio of early diastolic mitral ventricular filling velocity to early mitral annular velocity; eGFR = estimated glomerular filtration rate; ePASP = estimated pulmonary artery systolic pressure; HFpEF = heart failure with preserved ejection fraction; IVC = inferior vena cava; LDL = low-density lipoprotein; LVEDD = left ventricle end-diastolic diameter; LVEF = left ventricular ejection fraction; LVESD = left ventricle end-systolic diameter; LVOT VTI = left ventricular outflow tract velocity time integral; NICM = nonischemic cardiomyopathy; NT-proBNP = N-terminal pro-B-type natriuretic peptide; RV S′ = lateral tricuspid annulus peak systolic velocity; SOFA = sequential organ failure assessment; TAPSE = tricuspid annular plane systolic excursion; TR = tricuspid regurgitation; WRF = worsening renal function.

[a] – CAD was defined as: obstructive coronary artery lesions according to previous coronary angiogram, previous coronary artery stenting, previous coronary artery bypass surgery.

[b] – Signs/symptoms: dyspnea (1 point), oedema (absence of oedema – 0 points, ankles – 0.33 points, up to knees – 0.66 points, above the knees – 1 point), jugular venous distension (1 point).

Values presented as median [25th-75th percentile], mean ± standard deviation, or as *n* (%).
